# Editorial: Current state of postural research - Moving beyond the balance platform

**DOI:** 10.3389/fneur.2024.1362720

**Published:** 2024-01-23

**Authors:** Emily A. Keshner, Joyce Fung, Tanvi Bhatt

**Affiliations:** ^1^Department of Health and Rehabilitation Sciences, Temple University, Philadelphia, PA, United States; ^2^School of Physical and Occupational Therapy, McGill University, Montreal, QC, Canada; ^3^Feil/Oberfeld/CRIR Research Centre, Jewish Rehabilitation Hospital, Montreal, QC, Canada; ^4^Department of Physical Therapy, University of Illinois at Chicago, Chicago, IL, United States

**Keywords:** balance, gait, cortical processing, attention, emotion, perception

Assessment of postural control with a dynamic Balance Platform was a disruptive technology at its time (1). Findings emerging from research with the dynamic platform significantly altered not only the approach to studying control of posture, but also shaped theories of postural control over subsequent decades. In this Research Topic, Keshner et al. argue that the most dominant paradigm shift since adoption of the posture platform was that reactive postural control is not simply a reflexive process but one that is highly dependent on the demands of the specific task as well as the state of the performer. Hall et al. review evidence for a strong linkage between emotional state and the somatic nervous system in the control of posture. Our prior Research Topic (2) focused on postural research that challenged the view of postural control as a reflexive mechanism. The aim of this Research Topic was to solicit articles that focused on the concept of posture control as a complex and adaptable motor act and were not necessarily reliant upon the dynamic balance platform as an experimental protocol.

The contribution of cortical processing to posture control is an emergent theme in several of the papers in this Research Topic. Although underlying neural mechanisms that regulate postural stability are not yet well delineated, Fischer et al. hypothesized that shifts in attention contribute to inconsistencies in motor behavior by demonstrating increases in sample entropy as a measure of postural sway regularity under threatening circumstances. Through simultaneous EEG and EMG analyses, Stokkermans et al. identified distinct gain changes in the theta, alpha, beta, and low/high-gamma frequencies during the reactive balance responses for all leg muscles. Kannan et al. employed fMRI technology to identify significant associations between reactive balance control and cortico-subcortical regions even in individuals with mild cognitive impairment.

The finding of downstream control of postural behaviors does not in any way negate the importance of the sensory signals guiding postural responses. Sutter et al. demonstrated that the signals emerging from skin deformations generated by forces and pressures exerted between the foot skin and the standing surface increased sensory flow to the somatosensory cortex and improved balance control. Additionally, sensory flow to somatosensory cortex increased as did gamma activity over centroparietal regions during the preparation phase of the body weight transfers. Cleworth et al. looked specifically at perceptual thresholds at the ankle when facing a height-induced threat to balance and found raised perceptual thresholds and perceptions of increased motion of the foot suggesting cortical modulation of sensory feedback during threat induced instability.

Although training of automatic postural reactions has not proven robust in the treatment of instability and falls (3), findings that support the participation of cortical processes in planning and preparation for postural responses warrant attention in promoting experience-dependent neuroplasticity (4). Fadillioglu et al. provide evidence of the potential for training to improve postural reactions. Lockhart et al. found that training on a split-belt treadmill during gait and static postural stability tasks improved balance and stability of individuals with Parkinson's Disease. They conclude that these participants used their experiences with perturbation training to integrate and adapt feedforward and feedback behaviors to reduce falls, thereby illustrating that postural reactions practiced within a functional context can be learned.

Wang et al. explored a substantial component of cortically controlled behavior known as perceptual motor style. They quantified the variability that emerges both within and across individual performers and identified both static and dynamic markers that could specify the perceptual motor style. Significant heterogeneity was found across individuals performing locomotion in a height threatening virtual environment. Other markers of asymmetry in gait were explored in the lateral plane of motion, Dusane et al. showed that the ability to control lateral COM motion during walking could be a contributing factor to imbalance in people with incomplete spinal cord injury. Peterka et al. identified four major gait asymmetries (involving step width, ankle torque, stance duration and swing duration) as determinants of a separate mechanism in the control of mediolateral stability in gait. The variability of these asymmetry measures is shown to better distinguish between young and old performers than conventional measures associated with poor balance and fall risk.

Results from the research presented in this issue provide further evidence to support a significant contribution of both cortical and subcortical processing, attention control networks, and sensory-motor perceptual systems to the control of posture and balance under both perturbed and unperturbed (static and dynamic) conditions. The work done initially by Nashner and colleagues has, in fact, laid the foundation to a better understanding of control of posture and gait, using newly emerging technologies (e.g., mobile imaging and wearable sensors) and commercially available equipment that mimics the dynamic platform paradigm (i.e., motorized treadmills). The adoption of these approaches to postural control research in no way negates the continued value of the dynamic platform as a crucial paradigm that could be used both in stance and gait to examine cortical control (Keshner et al.). Instead, the evidence expands the importance of the study of reactive balance control by considering the contribution of cortical networks to posture and balance.

## Author contributions

EK: Writing – original draft, Writing – review & editing. JF: Writing – review & editing. TB: Writing – review & editing.

## References

[1] Nashner LM. Fixed patterns of rapid postural responses among leg muscles during stance. Exp Brain Res. (1977) 30:13–24. 10.1007/BF00237855 590411

[2] Keshner EA Fung J. Editorial: Current state of postural research – beyond automatic behavior. Front Neurol. (2019) 10:1160. 10.3389/fneur.2019.01160 31736864 PMC6834784

[3] Shapiro A Melzer I. Balance perturbation system to improve balance compensatory responses during walking in old persons. J Neuroeng Rehabil. (2010) 7:32. 10.1186/1743-0003-7-32 20630113 PMC2911463

[4] Kleim JA Jones TA. Principles of experience-dependent neural plasticity: implications for rehabilitation after brain damage. JSLHR. (2008) 51:S225–39. 10.1044/1092-4388(2008/018) 18230848

